# Disparities in cervical screening participation: a comparison of Russian, Somali and Kurdish immigrants with the general Finnish population

**DOI:** 10.1186/s12939-018-0768-2

**Published:** 2018-05-04

**Authors:** Esther E. Idehen, Päivikki Koponen, Tommi Härkänen, Mari Kangasniemi, Anna-Maija Pietilä, Tellervo Korhonen

**Affiliations:** 1 Institute of Public Health and Clinical Nutrition, Faculty of Health Sciences, University of Eastern Finland, Yliopistoranta 1, P. O. Box 1627, 70211 Kuopio, Finland; 2 Department of Public Health Solutions, National Institute for Health and Welfare (THL), Helsinki, Finland; 3 Department of Nursing Science, Faculty of Health Sciences, University of Eastern Finland, Kuopio, Finland; 4 Department of Public Health, Faculty of Medicine, University of Helsinki, Helsinki, Finland

**Keywords:** Cervical cancer, Cervical screening, Equity, Disparities, Healthcare service, Health inequities, Immigrants, Pap test

## Abstract

**Background:**

Cervical cancer is currently ranked as the fourth commonly diagnosed cancer in women globally. A higher incidence has been reported in low- and-middle-income countries, and the disease poses significant public health challenges. Evidence suggests that this disease is preventable by means of regular screening using the Papanicolaou (Pap) test. However, limited knowledge exists about disparities in cervical screening participation among immigrants compared with non-immigrants, in countries with universal cervical screening programmes. We aimed to examine disparities in cervical screening participation among women of Russian, Somali, and Kurdish, origin in Finland, comparing them with the general Finnish population (Finns). We controlled for differences in several socio-demographic and health-related variables as potential confounders.

**Methods:**

We employed data from the Finnish Migrant Health and Well-being Study 2010–2012 and the National Health 2011 Survey. Data collection involved face-to-face interviews. Data on screening participation in the previous five years from women aged 29–60 were available from 537 immigrants (257 Russians, 113 Somalis, 167 Kurds) and from 436 Finns. For statistical analyses, we used multiple logistic regression.

**Results:**

Age-adjusted screening participation rates were as follows: Russians 79% (95% CI 72.9–84.4), Somalis 41% (95% CI 31.4–50.1), and Kurds 64% (95% CI 57.2–70.8), compared with 94% (95% CI 91.4–95.9) among Finns. After additionally adjusting for socio-demographic and health-related confounders, all the immigrant groups showed a significantly lower likelihood of screening participation when compared with Finns. The Odds Ratios were as follows: Russians 0.32 (95% CI 0.18–0.58), Somalis 0.10 (95% CI 0.04–0.23), and Kurds 0.17 (95% CI 0.09–0.35). However, when additionally accounting for country of origin-confounder interactions, such differences were attenuated.

**Conclusions:**

Our results indicate disparities in screening participation among these immigrants and a lower likelihood of screening participation compared with the general Finnish population. To improve equity in cervical cancer screening participation, appropriate culturally tailored intervention programmes for each immigrant group might be beneficial.

## Background

Cervical cancer is currently ranked as the fourth commonly diagnosed cancer in women globally [1, 2]. A higher incidence of the disease has been reported in low- and-middle-income countries (LMICs) [1]. Worldwide, approximately 528,000 new cases of cervical cancer and 266,000 deaths resulting from the disease were reported in 2012. Nearly 90% of that mortality was in LMICs [1, 3]. Considerable evidence proves that the Papanicolaou (Pap) test is an effective screening tool for early detection of “pre-cancerous lesions,” with a follow-up procedure and a better prognosis [3–5]. Therefore, cervical screening and follow-up are recommended for all asymptomatic women [5, 6], irrespective of cultural background. The incidence of, and mortality resulting from cervical cancer have substantially declined in most developed countries [3–5, 7, 8]. However, the use of this healthcare service in some countries offering universal cervical screening involves significant racial or ethnic disparities [9–19]. This reflects that having the right, or equal access, to healthcare services, such as cervical screening, does not guarantee equal utilisation of the service [12, 20].

It is essential to explore disparities in cervical screening participation among various immigrant groups and non-immigrants, even when universal screening exits. Previous studies have revealed a higher risk of cervical cancer among women who seldom participate in screening or those not screened, compared with regularly screened women [21, 22]. Notably, studies have reported a higher risk of the disease among some immigrant groups compared with non-immigrants in several countries offering a universal screening programme [4, 11, 12, 16, 23].

Previous international studies have indicated that some socio-economic and health-related determinants are associated with non-adherence to screening recommendations. Thus, differences in these determinants would indicate additional potential disparities, which may confound disparities observed in cervical screening. Such determinants include a low educational level, unemployment, being unmarried, limited language proficiency, lack of knowledge of screening, and previous unpleasant screening experiences [9, 13, 17, 18, 23–26]. Other determinants are related to cultural beliefs and health-seeking behaviour found among some ethnic groups [14, 19, 27–29]. Additionally, migration-related issues have been highlighted as significant predictors associated with disparities in the screening participation. For example, younger age at migration, longer length of stay in the host country, and a higher level of acculturation, are determinants for a higher likelihood of screening participation among some immigrant groups [10, 26, 30–33]. Barriers to screening participation include limited understanding of screening and low level of education in the country of origin before migrating to the host country. Inability to navigate within the healthcare system and difficulties in obtaining health information are also barriers [24, 31, 33, 34].

Global mobility as a result of various factors, such as employment, war crises, education, and family reunion, raised international migrant numbers to 244 million in 2015. Approximately half (52.4%) of these migrants are women; [35] the migrant European population has also grown, with about 76 million migrants [36]. People from different cultures might have different expectations based on their health values and beliefs dependent on cultural norms [37]. These might influence their use of healthcare services, such as cervical screening participation. More recently, the health status of some immigrant groups referred to as “vulnerable populations” as compared with that of non-immigrants has drawn attention in the European public health sector [20, 38]. As such, great concern exists that immigrants referred to as “hard to reach” might lack access to healthcare services, such as screening, compared with the native population [26, 33]. Hence, the World Health Organisation and the European Commission have set equity in healthcare as a top priority goal in its member states, to address inequity in healthcare and to improve healthcare service practices and delivery [20, 38–40].

Finland has witnessed a substantial growth in its immigrant population, including women, in recent years. According to Statistics Finland, the immigrant population increased by 21% in 2016, compared to a year earlier [41]. Finland has offered organised mass cervical screening programme since the 1960s, using a five-year interval among women aged 25–65 [42, 43]. Personal screening invitation letters are sent to all eligible women, including immigrants, identified from the Finnish national population register [43]. In addition, women in this age group can participate in other (opportunistic) screening tests offered, or referred to, by their physicians or other healthcare providers [44]. Hence, in Finland, the effectiveness of cervical screening is well established [3, 7, 8, 45]. For instance, cervical screening coverage among those invited to mass screening is approximately 70% [43], and screening coverage among the general Finnish population is nearly 90% [44]. The incidence of cervical cancer is about 4/100,000, and mortality resulting from cervical cancer is around 1/100,000 women [43].

A previous study in Finland revealed relatively low screening participation rates as well as differences in, and significant barriers to cervical screening among some immigrant groups [9]. However, knowledge is limited about potential disparities in the screening participation among different population groups compared with the general Finnish population.

In this study, we aimed to examine disparities in self-reported cervical screening (Pap test) participation among women of Russian, Somali, and Kurdish, origin in Finland, compared with the general Finnish population (hereafter, Finns). We controlled for differences in several socio-demographic and health-related variables as potential confounders. With the increasing population of various immigrant groups, it is essential to explore disparities in cervical screening participation and identifying groups at risk of the disease. This study will potentially contribute to the existing knowledge of cervical screening participation among various immigrant groups. Additionally, we hope to aid policy making and to develop effective screening programmes tailored to immigrants and appropriate healthcare resource allocation, both at national and international levels.

## Methods

### Study population

We employed data from the Finnish population-based Migrant Health and Well-being Study (Maamu) 2010–2012 [46] and the National Health 2011 Survey [47] conducted by the Finnish National Institute for Health and Welfare (THL). The Maamu study sample consists of 3000 immigrants: 1000 from Russia or the former Soviet Union, 1000 from Somalia, and 1000 from Iraq or Iran (Kurdish background) [46]. These immigrant groups represent various geographical regions and reasons for migration. Among foreign language speakers in Finland, Russians are the largest group and Somalis the fourth largest; Kurdish Sorani speakers comprise the sixth largest group. Recently, Kurds from Iraq and Iran have been among the major groups in the quota of refugees accepted into Finland [48]. Our Somali group comprises mainly refugees and those women who came to Finland for reuniting with their families.

A random sample was drawn from the Finnish National Population Registry and stratified by municipality and participants’ country of origin [46]. The inclusion criteria were as follows: country of birth (Russia/Soviet Union, Somalia, Iraq or Iran) and mother tongue, as recorded in the population register. We included Russians (speaking Russian or Finnish) and Kurds (speaking Kurdish Sorani), the latter born in Iraq or Iran. Other inclusion criteria were age 18–64, living in one of the six Finnish cities with high proportions of immigrants (Helsinki, Espoo, Vantaa, Tampere, Turku, and Vaasa), and at least one year of stay in Finland.

Among the 3000 persons (1000 from each immigrant group) invited to the survey, the minimum participation rates for any one part of the study protocol were as follows: Russians 70% (*n* = 702), Somalis 51% (*n* = 512), and Kurds, 63% (*n* = 632). The study protocol included a face-to-face interview as well as a health examination and a health interview, with an option for a short interview for those unable or unwilling to participate in the longer health examination or interview. We analysed 537 responses on self-reported cervical screening participation in the previous five years; we obtained these from immigrants (257 Russians, 113 Somalis, 167 Kurds) aged 29–60, the recommended age range for the Finnish mass screening programme [43]. The Finnish reference group comprised participants from the National Population-based Health 2011 Survey, who lived in the same six cities as the participants in the Maamu survey. Approximately, 71% of them participated in at least one part of the study (*n* = 1582) [47]; from this reference group, we had 436 respondents.

### Data collection methods

The Maamu study comprises data from structured face-to-face interviews conducted by trained bilingual interviewers who used the native languages of the participants. The interviews consisted of questions on socio-economic- and health-related issues, including cervical screening participation. The Health 2011 survey data were collected using face-to-face interviews and self-administered questionnaires.

### Measures and variables

Based on the Finnish recommendation for the organised screening programme, the dependent variable in the study was self-reported cervical screening (Pap test) participation in the previous five years. As part of the health interview, participants were asked about any Pap test taken, including tests in the organised screening programme and in non-organised settings, such as private clinics (opportunistic tests); the answers were recorded as ‘yes’ or ‘no.’

Based on earlier studies indicating determinants associated with screening participation [10, 13, 14, 18, 23, 26, 29], our analyses considered differences in several potential confounders. We selected several potential confounders in the socio-demographic category: age, level of education, marital status, number of household members, employment status, residential area, and income sufficiency. Health-related confounders included self-perceived health status and leisure-time physical exercise; we asked them whether they had had a chronic illness, abortion, or miscarriage; participants also responded to whether they were obese (BMI > 30) and to whether they had ever smoked or given birth.

### Statistical analyses

For statistical analyses, we used the StataSE/13 software package. All analyses used finite population correction (FPC), with the effects of non-response corrected with Inverse Probability Weighting (IPW) [49]. The logistic regression model used for IPW was based on register information on the immigrant groups, age group, gender, city, and marital status. The weights were calibrated using the population size of each stratum. Using predictive margins, we calculated the adjusted prevalence estimates [50]. First, we analysed the main descriptive characteristics of the study population, i.e., weighted and age-adjusted prevalence (%) estimates for categorical variables and mean, plus standard error (SE) for the continuous variable (age). Further, we applied multiple logistic regression models to explore the statistical significance of the disparities in screening participation among these immigrant groups compared with Finns, the reference group (OR = 1.00). Model 1 was adjusted for only age while model 2 was additionally adjusted for the main effects of selected socio-demographic and health-related confounders. Then we tested country of origin-confounder interactions on screening participation, with *p* < 0.15 as a threshold. We included those country of origin-confounder interactions in Model 3, which was the final model based on the analyses.

## Results

### Descriptive results

Age-adjusted screening participation rates were as follows: Russians 79% (95% CI 72.9–84.4), Somalis 41% (95% CI 31.4–50.1), and Kurds 64% (95% CI 57.2–70.8), compared with 94% (95% CI 91.4–95.9) among Finns. Table 1 displays the descriptive characteristics of the study participants.

**Table 1:** Characteristics of participants by country of origin: weighted and age-adjusted proportions (%)^a,b^

Characteristics			Finnish (*n* = 436)		Russian *(n* = 257)		Somali (*n* = 113)		Kurdish (*n* = 167)	
Age: Years Mean (SE)			44.9 (0.46)		44.8 (0.61)		40.8 (0.81)		40.3 (0.56)	
Variable	Description	Total *n* of respondents	n	%	n	%	n	%	n	%
Pap test taken in the past five years	Yes	973	405	93.6	198	78.6	39	40.8	106	64.0
Education	High school in any country	971	313	74.8	218	87.6	14	15.6	68	43.9
Marital status	Married or cohabiting	970	305	73.9	170	67.0	84	78.8	129	75.4
Household members	Above three members	973	117	36.0	55	26.6	83	77.0	92	55.4
Employment status	Employed	970	359	79.8	143	51.7	16	15.1	55	32.9
Residential area	Outside metropolitan areas	973	118	24.9	89	16.4	31	9.4	67	43.2
Sufficient income	Yes	959	288	69.8	121	48.0	54	42.1	55	34.1
Self-perceived health status	Good	973	357	86.1	137	61.9	85	74.1	87	56.2
Ever given birth	Yes	928	293	60.9	223	79.9	99	92.8	157	92.4
Ever smoked	Yes	972	330	78.6	146	59.7	3	4.0	17	10.1
Obese	Body Mass Index (BMI) ≥30 kg/m2	962	91	16.6	54	17.5	49	42.2	47	27.6
Leisure-time physical exercise	Active	938	103	28.9	61	28.7	15	19.5	29	19.6
Any chronic illness	Yes	972	137	25.1	110	39.0	28	26.9	69	39.8
Any abortion	Yes	920	74	15.1	157	58.9	3	1.0	45	26.9
Any miscarriage	Yes	924	78	18.2	59	19.8	43	38.8	55	31.6

^a^For age, mean and Standard Error (SE)

^b^Among women who responded to the question of Pap test participation (*n* = 973)

### Screening participation dependent on country of origin-confounding variable interactions

Table 2 shows age-adjusted prevalence estimates of the screening participation based on the most essential confounding variables, which had significant interactions with the country of origin of these immigrant groups. Married Finns and Somalis participated in screening more often while Russians and Kurds tended to be the opposite. Employed Russians and Kurds participated more actively; employed Finns and Somalis, however, tended to participate less actively. Russians and Somalis having one to three household members participated more, but the opposite was true for Kurds and Finns. Those living outside of the metropolitan areas of Finland had higher screening participation among all the groups, except for the Somalis (Table 2).

**Table 2:** Age adjusted prevalence of Pap test participation by confounding variables with country of origin interactions

Variables	Finnish	Russian	Somali	Kurdish
	%	%	%	%
Marital status				
Married or cohabiting	96.5	73.7	46.4	63.7
Others^a^	87.5	78.8	38.8	66.5
Employment status				
Employed	91.2	82.5	38.7	73.8
Not employed	94.7	68.9	49.2	55.2
Household members				
One-three members	92.5	76.7	46.6	58.8
Above three members	94.9	73.4	38.0	76.3
Residential areas				
Metropolitan areas^b^	92.4	73.5	53.9	59.9
Other cities/outside of metropolitan areas^c^	95.1	81.3	14.5	78.5

^a^Including the singles, separated or divorced, and widow

^b^Metropolitan areas (Helsinki, Espoo, and Vantaa)

^c^Other cities (Tampere, Turku and Vaasa)

Table 3 displays the Odd Ratios [ORs] and 95% Confidence Intervals [CIs] of three logistic regression models for screening participation among the immigrant groups compared with the reference group (Finns’ OR = 1.00). When adjusted for only age, the likelihood of screening participation among all the immigrant groups was significantly lower. Compared with the reference group, Somalis had the lowest odds (Model 1). After adjusting for age and socio-demographic and health-related confounders, these disparities remained significant compared with the reference group; all our immigrant groups demonstrated a significantly lower likelihood of screening participation (Model 2).

**Table 3:** Logistic regression models for Pap test participation: Odds Ratios (OR) and 95% Confidence Intervals (CI)

Variables		Main effects	Finnish	Russian	Somali	Kurdish	*P*
			OR	OR (95% CI)	OR (95% CI)	OR (95% CI)	
Model^1^		OR (95% CI)	1.00	0.25 (0.15–0.40)	0.05 (0.03–0.08)	0.12 (0.07–0.19)	
Model^2^	Description	OR (95% CI)	1.00	0.32 (0.18–0.58)	0.10 (0.04–0.23)	0.17 (0.09–0.35)	
Education	High school in any country	1.65 (1.09–2.50)					
Marital status	Married or cohabiting	1.27 (0.80–2.00)					
Household members	Above 3 members	1.35 (0.83–2.19)					
Employment status	Employed	1.68 (1.11–2.55)					
Residential areas	Outside metropolitan	1.72 (1.14–2.58)					
Sufficient income	Yes	1.36 (0.90–2.07)					
Self-perceived health status	Good	1.32 (0.80–2.18)					
Ever given birth	Yes	1.13 (0.62–2.06)					
Ever smoked	Yes	0.92 (0.55–1.53)					
Obese	BMI > 30Kg/M^2^	0.83 (0.54–1.26)					
Leisure-time physical exercise	Active	1.53 (0.93–2.51)					
Any chronic illness	Yes	1.66 (1.03–2.66)					
Any abortion	Yes	1.05 (0.66–1.66)					
Any miscarriage	Yes	1.19 (0.76–1.86)					
Model^3^			1.00	0.74 (0.16–3.39)	0.34 (0.05–2.49)	0.23 (0.05–1.10)	0.1642
				Interaction terms			
Marital status	Married or cohabiting*	4.58 (1.92–10.9)	1.00	0.16 (0.05–0.51)	0.32 (0.06–1.65)	0.18 (0.05–0.63)	0.0103
Employment status	Employed*	0.46 (0.15–1.44)	1.00	4.91 (1.30–18.6)	1.21 (0.20–7.48)	6.07 (1.50–24.6)	0.0225
Household members	Above 3 members*	1.47 (0.38–5.64)	1.00	0.56 (0.12–2.63)	0.48 (0.08–2.93)	1.66 (0.37–7.53)	0.1841
Residential areas	Outside metropolitan*	1.53 (0.57–4.15)	1.00	1.05 (0.30–3.68)	0.08 (0.01–0.57)	1.77 (0.52–6.01)	0.0130
Ever smoked	Yes	3.15 (1.39–7.16)	1.00	0.25 (0.08–0.75)	N/A	0.13 (0.03–0.58)	0.0091
Education	High school	1.79 (1.16–2.77)					
Sufficient income	Yes	1.50 (0.97–2.30)					
Self-perceived health status	Good	1.33 (0–79-2.25)					
Ever given birth	Yes	1.06 (0.55–2.04)					
Obese	BMI > 30Kg /M^2^	0.81 (0.52–1.25)					
Leisure-time physical exercise	Active	1.50 (0.88–2.57)					
Any chronic illness	Yes	1.64 (0.98–2.73)					
Any abortion	Yes	0.98 (0.60–1.61)					
Any miscarriage	Yes	1.29 (0.81–2.06)					

^1^Adjusted for age

^2^Adjusted for age, education, marital status, employment status, family size, residential area, sufficient income, self-rated health status, ever given birth, obesity (BMI), physical exercise, any chronic illness, any abortions, any miscarriage, and ever smoked

^3^Adjusted for age, education, marital status, employment status, household members, residential area, sufficient income, self-rated health status, ever given birth, obesity (BMI) physical exercise, any chronic illness, any abortions, any miscarriage, ever smoked. Including study group Interactions with: Marital status, employment status, residential areas, household members and ever smoked

*N/A* not available due to no smokers in this group

*Interaction *p*-values (Interaction –test results)

Model 3 provided the results adjusted for the direct effects of multiple confounders and included country-confounder interactions. Those confounders which had major effects were age, socio-demographic (education and income) and health-related variables. Health-related confounders included self-perceived health status and leisure-time physical exercise; we asked them whether they had had a chronic illness, abortion, or miscarriage; participants also responded to whether they were obese (BMI > 30) and to whether they had ever smoked or given birth. Country of origin and the following confounding variables exhibited significant interaction: marital status, employment status, number of household members, residential area, and whether they had ever smoked.

When we adjusted the final model simultaneously for these confounders and interactions, compared with the reference group, we still detected lower OR point estimates of screening participation among all these immigrant groups. However, none of the ORs remained statistically significant, as indicated by the 95% CIs which include 1 (Table 3).

## Discussion

The rationale behind cervical screening using the Papanicolaou (Pap) test is early detection of “pre-cancerous lesions” and effective treatment of abnormalities [3–5]. However, full compliance with screening recommendations is essential to achieve this goal. This population-based study examined disparities in cervical screening (Pap test) participation among women of Russian, Somali, and Kurdish, origin residing in Finland and compared it with the general Finnish population (Finns), our reference group. In our analyses, we accounted for country of origin differences in multiple determinants of cervical screening were considered as potential confounders. We also accounted for multiple country of origin-confounder interactions. The lower odds for screening participation compared with the Finns indicated significant disparities among the immigrant groups studied. However, such disparities were attenuated when accounting for socio-demographic and health-related confounders specific to each immigrant group. Thus, we demonstrated that multiple screening determinants specific to each country of origin might underlie the disparities initially observed.

Although Finland offers universal cost-free cervical screening to all eligible women, including legal immigrants, our findings demonstrate disparities among these immigrants and lower screening participation in comparison with the Finns. Our results are consistent with previous international studies showing disparities in screening participation among various immigrant groups in countries offering universal screening programmes [10–13, 16–18, 23]. Although such disparities have been widely reported world-wide, to the best of our knowledge, similar disparities in Finland have not been published earlier.

The main goal of universal healthcare is that “all people can obtain the health services they need without suffering financial hardship” [51]. This comprises the right of access to healthcare services, such as cervical screening. However, disparities in cervical screening practices could lead to underutilisation of the screening service and thus increase the incidence of, and mortality from, the disease [52]. Earlier studies have highlighted the higher risk of the disease among non-screened participants or those who seldom participate in the screening, compared to regularly screened women [4, 16, 21]. This feature exists especially among some immigrant groups, compared with the native population [4, 11, 12, 16, 23]. With immigrant population increasing, disparities that persist will increase national healthcare expenditure [52].

When observing such disparities, it is relevant to ask why some immigrant women have lower adherence to recommendations related to women’s health. According to previous studies, non-adherence to screening recommendations among some immigrant groups has associations with lack of knowledge about screening, lack of disease symptoms, and religious faith. Not having a female healthcare professional to perform the screening test may also function as a barrier to screening participation [13, 14, 25, 27–29, 53]. Based on an earlier study [54], perceptions about, or differences in healthcare service practices or delivery in the country of origin of the immigrant and his/her host country might also contribute to the problem. Participants in this particular study expected their healthcare providers to take an active role in preventive healthcare advice or recommendations as it was practised in their country of origin. Two studies have demonstrated the role of healthcare providers and personal physicians; providing referrals for screening increases the likelihood of screening participation [4, 55]. Migration-related issues, namely, longer period of stay in the host country, younger age at migration, and higher level of acculturation, are determinants for screening participation among some immigrant groups [14, 26, 31–33]. However, in this study we did not adjust for these determinants, because our main focus was in comparison with the general Finnish population, where such variables are irrelevant.

In our study, overall, among these immigrant groups, the highest screening participation rate was observed among Russians followed by the Kurds while the Somalis had the lowest participation rate; these results are consistent with those of an earlier study [9]. These population groups have migrated from different continents and countries; thus, their cultural beliefs, norms, health beliefs, religion, ethnicity, and health-seeking behaviours, might vary [13, 14, 53, 54, 56].

The relatively active screening participation among Russian women compared with other immigrant women might be partly due to their higher level of language proficiency, with about 90% understanding Finnish or Swedish, the official languages of Finland, as demonstrated in an earlier study [9]. Understanding the official language of the host country increases the likelihood of screening participation [9, 13, 23, 25, 33, 55]. Adequate language skills enable the individual to navigate within the healthcare system for obtaining health promotion information, such as the importance and aims of the screening. Language skills also facilitate effective communication with healthcare officials [9, 32, 33, 55].

We adjusted our analyses for differences in multiple confounders, which may at least partly explain some of the disparities initially observed. For example, the association between higher screening participation and being married or cohabiting, although found only among Finns and Somalis, could explain the essential role of the husband or partner in screening participation, consistent with a previous study [53]. Further, the low screening participation found among Somali or Muslim women might be related to unfamiliarity with screening due to the unavailability of the screening programme, lack of understanding of healthcare practices, or lack of screening affordability, in their country of origin [57]. Additionally, some Somali women have poor language proficiency, low level of education, or both; thus, they are unable to obtain health information, as shown in previous studies [9, 24, 31, 33, 34]. The experience(s) of embarrassment associated with female genital mutilation (FGM) or anxiety associated with screening and unpleasant screening experience(s), or both, might influence them [24, 33]. Further, the lower screening participation rate among Somalis living outside the metropolitan areas of Finland might be related to difficulties in transportation. Similar features were presented in mammogram screening [19].

To increase cervical screening participation among these immigrant groups, promoting screening awareness, disseminating information and offering education in the immigrants’ native language, as well as providing interpreters and female health professionals, are vital. Organising transportation when possible might also be helpful [24, 28, 29, 33, 34, 56, 58].

## Strengths and limitations of the study

This study has a population-based design and a reasonable response rate among Russian, Kurdish, and Finnish, participants. Bilingual interviewers who spoke and understood the native language of the participants themselves interviewed the participants; most were female. This feature enhances participant willingness and enables addressing gender-related issues. It also offered an opportunity to clarify research aims or questions raised during the interview [46].

The study also has some limitations. Responses to cervical screening participation based on retrospective self-reporting might involve some recall bias [59]. Response rates differed across immigrant groups. One explanation for this might be the differences in cultural backgrounds and beliefs; their reasons for migration are different. Refugees may be more concerned about data secrecy and may harbour more mistrust towards authorities. Furthermore, the particularly low survey response rate among Somalis might bias the results and limit the statistical power of the analyses. However, this limitation was statistically accounted for. Nonetheless, to the best of our knowledge, ours is the first study to analyse and report disparities in cervical screening participation among immigrant groups compared with the general Finnish population.

## Conclusions

Although Finland offers universal cervical screening to all eligible women, our results indicate disparities in screening participation among these immigrants and a lower likelihood of screening participation compared with the general Finnish population. To improve equity in cervical cancer screening participation, appropriate culturally tailored intervention programmes for each immigrant group might be beneficial. Further research is needed to enhance understanding of cultural and health beliefs, as well as of norms which may be specific to each immigrant group; we especially need qualitative studies.

## Abbreviations

FGM: Female genital mutilation
LMICs: Low- and- middle-income countries
